# Cost-effective fabrication, antibacterial application and cell viability studies of modified nonwoven cotton fabric

**DOI:** 10.1038/s41598-022-06391-5

**Published:** 2022-02-15

**Authors:** Rahat Nawaz, Sayed Tayyab Raza Naqvi, Batool Fatima, Nazia Zulfiqar, Muhammad Umer Farooq, Muhammad Najam ul Haq, Dilshad Hussain, Asghar Javeed, Azhar Rasul, Laila Jafri, Saadat Majeed, Waheed Qamar Khan

**Affiliations:** 1 Division of Analytical Chemistry, Institute of Chemical Sciences, Bahauddin Zakariya University, Multan, 60800 Pakistan; 2 Department of Biochemistry, Bahauddin Zakariya University, Multan, 60800 Pakistan; 3 International Centre for Chemical and Biological Sciences, HEJ Research Institute of Chemistry, University of Karachi, Karachi, 75270 Pakistan; 4 Department of Pathology, Nishtar Medical University, Multan, 60800 Pakistan; 5 Department of Zoology, Govt. College University Faisalabad, Faisalabad, Pakistan; 6 Department of Life Sciences, Abasyn University, Islamabad Campus, Islamabad, Pakistan; 7 Institute of Advanced Materials, Bahauddin Zakariya University, Multan, 60800 Pakistan

**Keywords:** Biochemistry, Chemistry, Materials science

## Abstract

In the present work, nonwoven cotton fabric was modified for antibacterial applications using low-cost and eco-friendly precursors. The treatment of fabric with alkali leads to the formation of active sites for surface modification, followed by dip coating with silver nanoparticles and chitosan. The surface was chlorinated in the next step to transform amide (N–H) groups in chitosan into N-halamine (N-Cl). The modified and unmodified surfaces of the nonwoven cotton fabric have been characterized by FTIR, SEM, and XRD. The active chlorine loading is measured with iodine/sodium thiosulphate. The antimicrobial activity and cell toxicity assay were carried out with and without modifications of nonwoven cotton fabric. The antimicrobial efficacies of loaded fabric were evaluated against four bacterial species (*Micrococcus luteus*, *Staphylococcus aureus*, *Enterobacter aerogenes,* and *E.coli*). It was found that modified fabric exhibited superior efficiency against gram-positive and gram-negative bacterial strains as compared to their bulk counterparts upon exposure without affecting strength and integrity of fabric. The overall process is economical for commercial purposes. The modified fabric can be used for antimicrobial, health, and food packaging industries, and in other biomedical applications.

## Introduction

A large number of health care applications have introduced recyclable antimicrobial wound dressings and biomedical fabrics^1^. Antimicrobial activity for a long period, stability, and fast disinfection capability with time are defining factors that increase the resistance properties of these materials against microbes^2^. The medical industry including carpets, beds, accessories, and other allied items are facing some serious issues of bacterial contamination of fabrics which may cause severe infections in patients and workers^3^. Hospital s' wearable accessories including lab coats and costumes are the main shields against bacteria, but these articles often do not offer sufficient antimicrobial properties^4,5^. Therefore, the modified inexpensive, biodegradable, rechargeable and recyclable, less toxic, durable, and eco-friendly antimicrobial wound dressings and fabrics are needed to be developed. This could potentially be achieved by incorporating stable and non-bleached *N-halamine* into the materials as biocides^6^. Such materials have been used owing to their good stability, regenerate ability, and high efficacy to inactivate bacteria^7^. Cotton cellulose-based materials are highly suitable objects for the growth of bacteria but can be functionalized in a facile way to enrich with growth inhibition properties. The wet finishing or grafting, layer by layer assembly, and dip coatings techniques are commonly used to produce a stable cotton fabric with enhanced antibacterial resistance properties^8,9^. The previous studies showed that *N-halamine* effectively forms a strong N-X (Br, Cl, I) bond through covalent interactions at the fabric surface and prevents the leaching of halogen atoms in an oxidative state of + 1. It is well known that the antibacterial action of *N-halamine*s is due to the positive halogens from *N-halamine*s that are transferred to suitable receptors in microbe cells. In this way, enzymatic or metabolic cell processes are inhibited and consequently killing bacteria and other parasites^10,11^. *N-halamine*s are promising functionalities that provide immediate bacterial inhibition against a broad range of microorganisms without affecting the external and internal environment of the host^10,12^. *N-halamine*s modification of fabric surfaces has been done by different methods including grafting^13^, layer by layer assembly^14^, and dip coatings^15^. *N-halamines* functionalization is carried out at nitrogen-containing surfaces containing imides, amides, or amines through halogenations (X = Br, Cl, I). The cotton cellulose modified with nitrogen-containing monomers and successive chlorination provides prompt inactivation against various bacterial strains^16–18^. Chitosan is a naturally existing polymer and has special characteristics like biodegradability; non-toxicity, cationic nature, and antimicrobial activity as well as incorporated with NH_2_ groups^19^^,^^20^. The NH_2_ groups in chitosan are transformed easily into *N-halamines* upon treatment by strong oxidants like household bleach. The treated fabric then acquires powerful antibacterial efficacy^21^. The aforementioned activity is due to the release of positive chlorine atoms upon dissociation of *N-halamines* on interaction with microbes^10,22^. The active chlorine contents thus released can be recharged and reloaded with chlorine from successive bleach treatments. The regeneration of surfaces is low cost and very simple. It has been reported that the biocidal efficiency of *N-halamine* materials largely depends upon the active surface area^23^. The large active surface area provides more active sites for the bactericidal activity to occur. Desirable surface area is obtained by incorporating various substrates like nanomaterials into dressings, fabrics, and packing materials along with *N-halamines*^24,25^. Nanomaterials not only inhibit or retard microbial growth but also improve the shelf-life of antimicrobial materials^26–28^. In nanotechnology, nanomaterials like silver, copper, and gold are distinctively important due to their special chemical and biological activities^29,30^. Metallic silver and silver-based nanomaterials are popular agents used for a long time in different health care products to enhance antibacterial effectiveness and to reduce infections^31,32^. Significantly at the nano-scale, silver materials show exceptional physical, chemical, biological properties than other bulk materials and are non-toxic^33^. Silver materials exists in different oxidation state (0, + 1, + 2, + 3). The antimicrobial activity of the silver substrates is dependent upon the bioavailability of silver in the + 1 state. The silver ions in the + 1 oxidation state slowly releases from the substrate and interact with microorganisms at the cellular level, releases and effectively damage cellular material^34^. The sufficient availability of silver ions, water solubility, and longevity of antimicrobial activity are key challenges and limitations. Currently, researchers have been focused to develop a highly active antimicrobial dressings by using *N-halamine* biocides. Different kinds of *N-halamine* compounds have been extensively investigated for antimicrobial activities.

Currently, researchers have been focused to develop a highly active antimicrobial dressings by using *N-halamine* biocides. Different kinds of *N-halamine* compounds have been extensively investigated for antimicrobial activities^35^. Inspired by earlier findings, this paper aims to investigate nonwoven cotton fabric coated with *N-halamine* and silver nanoparticles to enhance synergistically their effectiveness against microbial growth. *N-halamine*, chitosan, and silver nanoparticles modified nonwoven cotton fabric (hereafter termed and read as N*-halamine*-chitosan@AgNPs (NWCF)) are fabricated by the dip-coating method. This method of coating is considered facile, environment friendly requires fewer chemicals, and is cost-effective^36^. The dip-coating method is widely used in many industrial and laboratory scales methods for the coating of the surface of different substrates^37^. The chlorinated chitosan and silver nanoparticles nonwoven cotton fabric is evaluated for their antimicrobial activity. The fabric is also tested for cell toxicity assays against *Hela Cell* Lines. The main features of this study include simple synthesis methodology, low cost, enhanced antimicrobial properties due to a combination of three different materials (silver nanoparticles, chitosan and *n-halamine*). In addition, the lower toxicity of the modified fabric enhanced its application potential. The uniqueness of the present modified method is that fabric surfaces can easily be reactivated, modified, and reproduced by simply treating them with sodium hypochlorite. The fabric also shows stability for a longer time.

## Experimental

### Reagents, chemicals and softwares

Silver nitrate (AgNO_3_), Ascorbic acid (C_6_H_6_O_8_), Potassium hydroxide (KOH), and Sodium thiosulphate (Na_2_S_2_O_3_) were purchased from Merck. Sodium hypochlorite (12.5% solution), Hydrochloric acid (HCl), Potassium Iodide (KI), and Acetic acid (CH_3_COOH) were obtained from Sigma Aldrich. Nonwoven cotton fabric was obtained from a local market. Chitosan (C_56_H_103_N_9_O_39_) was purchased from Junsei Chemical Co. Ltd. Non-ionic detergent (Triton X-100) was used for fabric cleaning. All the statistical calculations and graphs were drawn using excel 2007 and OriginPro8 software. The images were modified in Image J and powerpoint 2007 softwares. Chemical Structures were drawn in Chemoffice 2004 software. Photographs of fabric were taken with Samsung Glaxy J7.

### Cleaning and activation of nonwoven cotton fabric

The fabric was obtained from the local market and was treated as the methods described earlier with slight modifications. 1 cm × 1 cm dimensions piece of nonwoven cotton fabric were cut, clean, and boiled in 50 mL hot water (100 °C) for 30 min using non-ionic detergent (Triton X-100). The fabric was then washed several times with hot water followed by cold water and dried in an oven at 90 °C. The dried fabric samples were stored at room temperature in desiccators and then used for further treatment and characterization.

For activation of fabric surface, pieces of dried nonwoven cotton fabric were soaked in 30 ml potassium hydroxide (6 M) solution for 30 min at room temperature with an occasional mechanical stirrer. The alkali-activated nonwoven cotton fabric was rinsed several times with distilled water, then padded through a laboratory wringer at low-pressure settings and kept in wet condition until the next treatment. The resulting modified non-woven cotton fabric was named and abbreviated as Nonwoven Cotton Fabric (NWCF).

### Surface modification of activated nonwoven cotton fabric with silver nanoparticles

Further surface modification of activated nonwoven cotton fabric with silver nanoparticles was carried out by following the previously reported method with slight modifications. Briefly, the activated nonwoven cotton fabric piece was immersed in freshly prepared 0.04 M silver nitrate solution, stirred for 30 min at room temperature. The fabric was taken out from the solution, washed thoroughly with distilled water several times to remove excessive unabsorbed silver ions. Silver ions on the fabric were then reduced using a reducing agent using 0.02 M ascorbic acid solutions and stirred again for 30 min at room temperature. The ascorbic acid solution turned clear to milky which indicated the formation of silver nanoparticles^38^. Silver coated nonwoven cotton fabric taken out, washed with distilled water, then padded through a laboratory wringer at low-pressure settings, dried at 65 °C for 90 min in the oven. The obtained dried fabric was labeled as silver nanoparticles modified nonwoven cotton fabric (AgNPs-NWCF). The control nonwoven cotton fabrics for comparisons were prepared under the same conditions without the addition of the silver precursor solution.

### Surface modification of AgNPs-Nonwoven cotton fabric with chitosan

The surface of AgNPs-NWCF with chitosan was modified by the dip-coating method^39,40^. Simply, 2 g of chitosan (Molecular weight:1526.5 g/ mol.) was added in 100 mL of 3 % CH_3_COOH aqueous solution at room temperature and vigorously stirred for 30 min to obtain a final 2 % clear solution of chitosan. AgNPs-NWCF was soaked in 2 % chitosan solution for 2 min. Similarly, the second coating was done in a fresh chitosan solution of the same concentration. Between each coating, fabric samples were washed with distilled water subsequently to remove unbound and loosely attached chitosan and were dried. Chitosan modified fabric samples were then passed through a laboratory wringer (Wringer TD110, Testex, Hong Kong) at low-pressure settings, oven-dried at 60 °C for 60 min. All the samples obtained were kept at 105 °C for 4 min, dried, and stored for further analysis. The samples prepared were termed as chitosan@ AgNPs-NWCF. The control fabrics were prepared with the same conditions without the addition of chitosan.

### *N-halamine*s formation on chitosan@ AgNPs-Nonwoven cotton fabric

*N-halamine* functionalities were created at the surface of chitosan modified AgNPs-nonwoven cotton fabric to obtain oxidizing halogen moiety i.e. chlorine, as described in earlier reports with slight modifications^40^. It is generally believed that amine groups in chitosan having N–H bond as precursor changed into N–X bond from *N-halamine* at the surface of the fabric. In the present study, modified cotton fabric was dipped into 10 mL of 10% household bleach (sodium hypochlorite solution 6% Cl^+^) solution at pH 7 for 30 min at room temperature. After chlorination, the fabric was rinsed with deionized water several times to remove any unbound and free chlorine from the fabric surface. The fabric was then dried at room temperature for 24 h. The modified fabric was taken for further analysis and characterization. The samples prepared were termed *N-halamine*-chitosan@ AgNPs-NWCF. The control fabrics were prepared with the same conditions without treating with sodium hypochlorite solution.

### Analysis of loaded chlorine contents on Chitosan@ AgNPs-Nonwoven cotton fabric (NWCF)

Loaded chlorine contents on all prepared samples were measured by using the iodine/ thiosulphate titration procedure^41^. Generally, 1 g of KI was added to 40 mL of CH_3_COOH (1_%_) containing 0.06 g (0.85 cm × 1.0 cm) of *N-halamine*-chitosan@ AgNPs NWCF, solution color does not change. The solution was stirred vigorously for one hour, the color changed slowly from colorless to light yellow. After one hour of stirring, KI solution containing *N-halamine*-chitosan@ AgNPs NWCF was titrated with 0.01 M sodium thiosulfate (Na_2_S_2_O_3_), until the endpoint (colorless). For comparison, the whole procedure was also performed for chitosan@ AgNPs NWCF and AgNPs NWCF without treating with chlorine. The final volume of sodium thiosulfate consumed during titration was calculated. The loaded chlorine concentration for modified and unmodified fabric samples was calculated by using the following equation (Eq. 1).

1 $$Cl\,\left(\%\right)= \frac{ 35.5}{2}\times \frac{\left(Vcl-V0\right)\times {10}^{-3}\times 0.01}{WCl}\times 100.$$

where 35.5 amu is the atomic mass of loaded chlorine. W_Cl_ weight (g) of the fabric piece, V_0_ and V_Cl_ are volumes of N_2_S_2_O_3_ solution consumed during titration of chlorine-treated and untreated nonwoven cotton fabric, respectively.

### Photostability and shelf-life of *N-halamine*-Chitosan@AgNPs-Nonwoven cotton fabric

The photosensitivity and shelf life of fabric samples, chitosan@ AgNPs**-**NWCF, chitosan@ AgNPs**-**NWCF, and *N-halamine*-chitosan @ AgNPs**-**NWCF with dimensions of 0.85 cm × 1.0 cm were exposed to a UVA lamp (345 nm) for 6 weeks at room conditions. The samples were investigated at different time intervals of 2, 4, and 6 weeks for loss of chlorine contents. Similarly, in another experiment samples were stored in dark under the same experimental conditions. The stability in terms of loss of chlorine contents and durability for all samples was measured using iodine/ thiosulphate titration. The percent loss in chlorine loading for all samples under light and dark was calculated and compared.

### Antimicrobial efficacy analysis

Tests were conducted to determine the antibacterial efficacy of modified non-woven cotton fabric samples as described earlier^42,43^. *Micrococcus luteus*, *Staphylococcus aureus* (AATCC (Test Method 100 of American Association of Textile Chemist and Colorists 6538) were used as a gram-positive bacterium, and *Enterobacter aerogenes, Escherichia coli* (O157: H7, AATCC 43,895) were used as a gram-negative bacterium. Generally, (0.85 cm × 1.0 cm dimensions) AgNPs- nonwoven cotton fabric (Control-I) was taken for analysis as described. Firstly, the bacterial suspension was prepared in phosphate buffer (pH 7) to generate a known population (Colony Forming Units, CFU). 25 µl of the bacterial suspensions containing 1.7 × 10^5^ CFUS was added to the center of AgNPs-nonwoven cotton fabric (Control-I). The bacterial colony was covered with second AgNPs-nonwoven cotton fabric (Control-I) of the same composition and dimension. After 5 min, the fabric piece was taken off, quenched with 5.0 mL of sterile 0.02 N sodium thiosulfate solution to remove and neutralize all oxidative chlorine. The samples were vortexed for 3 min to remove all bacteria from fabric surfaces. Bacteria-containing aliquots were diluted with 100 mL PBS (pH 7). Aliquots were transferred to 6 mm Trypticase soy agar disk plates, incubated at 37 °C for 24 h, and counted for viable CFU of bacteria. The same procedure was followed for the other two fabric pieces of (0.85 cm × 1.0 cm dimension) chitosan@AgNPs nonwoven cotton fabric (termed as Control-II) and *N-halamine*- chitosan@AgNPs -nonwoven cotton fabric (Sample). The bacterial inhibition growth analyses were carried out in triplicate. The experiments in this study were only conducted for the uptake of the bacteria and behavior of bacteria for the designed nonwoven cotton fabric. The removal of bacteria from fabric surface is not studied in the present work.

### Cell viability studies using MTT assay

MTT assay is done to evaluate the cell viability of all nonwoven modified fabric samples in HeLa cell lines culture following reported procedures^42–44^. The cells were grown as monolayer cultures in a DMEM medium. The cultures were kept at 37 °C in a humidified incubator (5 % CO_2_, 95 % air). All experiments were performed during the exponential phase of cell growth. The cells were grown in 96-well microplates for cell culturing at a concentration of 1 × 10^4^ cells/ well for 24 h. The culture medium was then removed and transferred with modified and unmodified nonwoven fabric. Samples of the viability of the cells in the presence of AgNPs-NWCF and chitosan@AgNPs-NWCF served as the negative control. The cell viability studies of the AgNPs-NWCF, chitosan@AgNPs-NWCF and N-halamine chitosan@AgNPs-NWCF against *HeLa cell* lines were evaluated using a colorimetric method following Mossman’s procedure. The cells were incubated for 3 h with 5 mg MTT (3-(4, 5-dimethylthiazol-2-yl)-2, 5-diphenyltetrazolium bromide) 10 mL DMEM solution ) at 37 °C (5 % carbon dioxide) in FBS free medium. The formed blue MTT formazan was extracted with a mixture of absolute ethanol and DMSO (1:1). The quantitative analysis was performed by absorbance measurements in an automatic microplate reader at 460 nm. The total cell viability is calculated by the following equation and expressed as a percentage of control.

2 $$Cell\,viability= \frac{\left({A}_{NWCF}-{A}_{blank}\right)}{\left({A}_{control}-{A}_{blank}\right)}\times 100 \%.$$

where *A* = absorbance measures at 460 nm. *A*_*NWCF*_ for modified and unmodified fabric samples, *A*_*control*_ for positive control and negative control. The values were calculated for triplicate measurements.

## Results and discussions

### Synthesis and characterization of modified Nonwoven fabric samples

**Figure 1:**
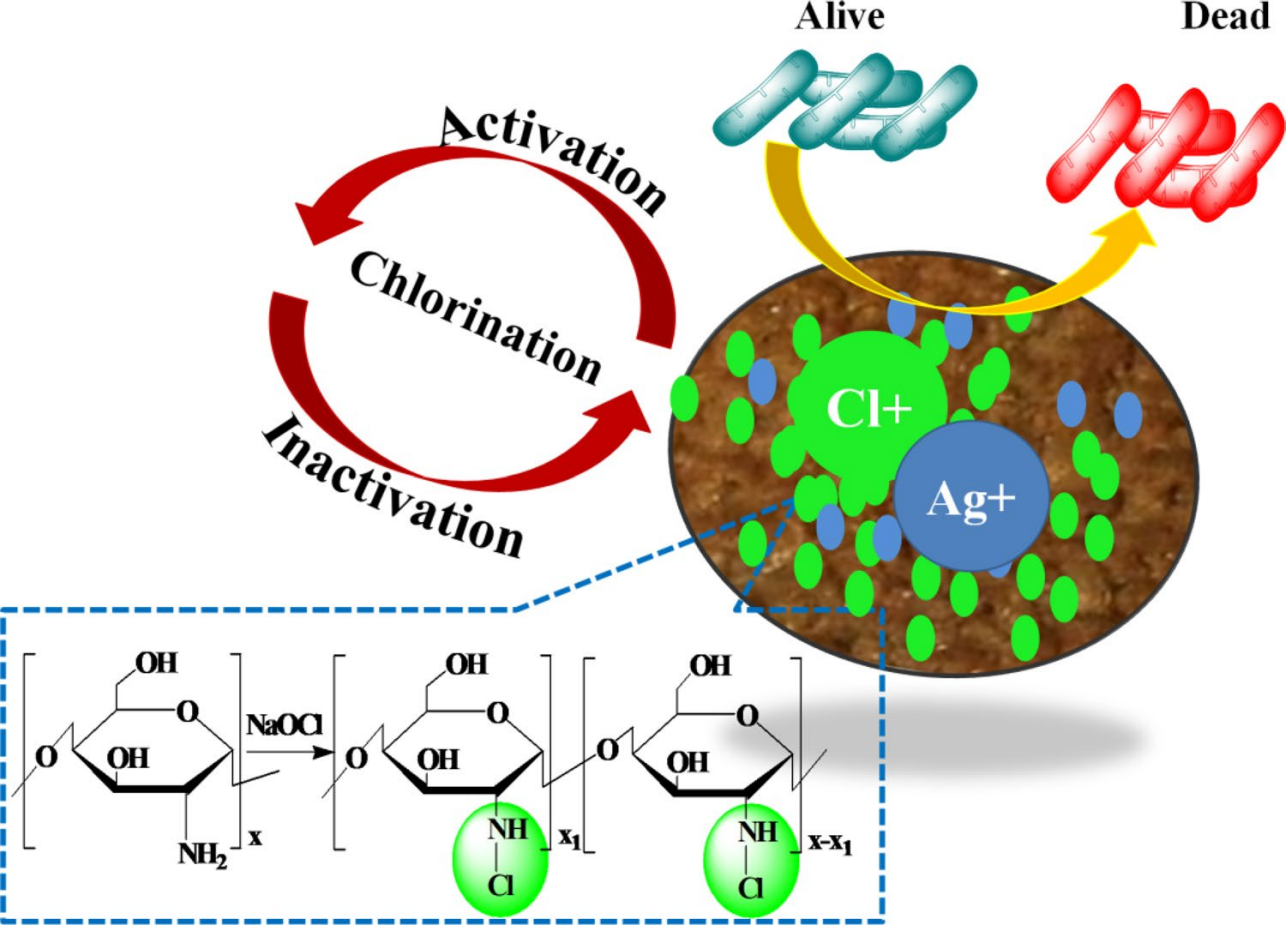
Graphical representation of modified nonwoven cotton fabric and antibacterial activity.

Samples of fabrics were modified following a three-step process, cleaning and activation of fabric surface, coating with silver nanoparticles, and N-halamine formation through chlorination of chitosan at fabric (Fig. 1).

**Figure 2:**
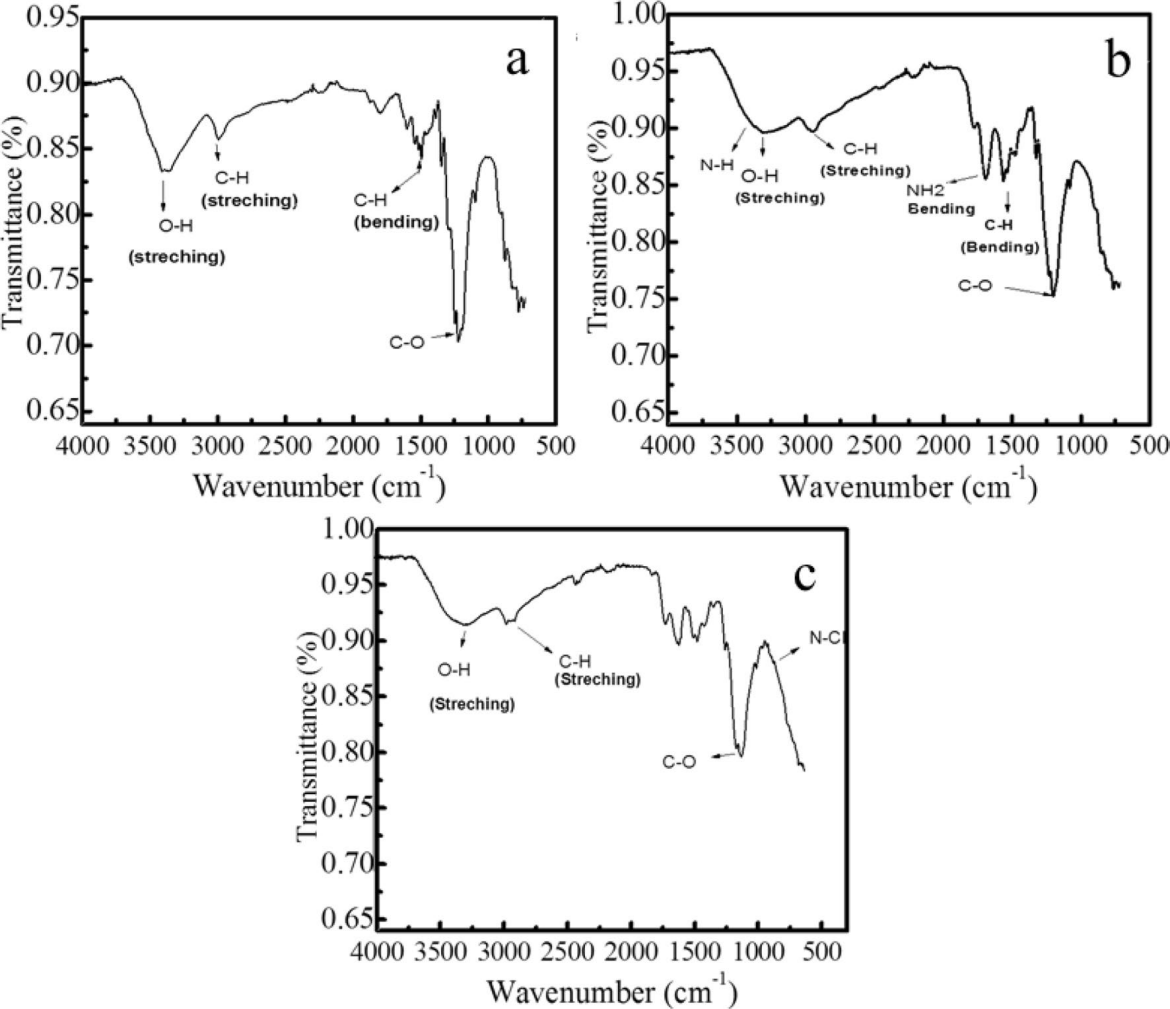
FTIR spectra of AgNPs-nonwoven cotton fabric (**a**), Chitosan@AgNPs nonwoven cotton fabric (**b**), and *N-halamine*-chitosan@AgNPs-nonwoven cotton fabric (**c**).

The modification was carried out simply by using the dip-coating method. All experiments were carried out at room temperature. The successful attachment of modifiers on non-woven cotton fabric was investigated further using different analytical techniques. Surface Characterization of nonwoven cotton fabric modified with AgNPs, chitosan@AgNPs, and *N-halamine*-chitosan@AgNPs was performed by FTIR (Fourier Transform Infrared) spectroscopy, SEM, and XRD. FTIR spectra (Fig. 2a) show characteristic peaks for AgNPs-nonwoven cotton fabric at 3348 cm^−1^ for O–H bonds (stretching vibration), at 2910 cm^−1^ for C–H bond (stretching vibration), and at 1313 cm^−1^ (C–H bending vibration). The peak due to the C–O bond is intense and broad, shifts to 1250 cm^−1^ because of AgNPs coated on nonwoven cotton fabric. These results reveal the bonding of silver nanoparticles with O-atoms of cellulose of the fabric. Coating of chitosan on the surface of AgNPs-nonwoven cotton fabric was also characterized by FTIR. Peaks at 3278 cm^−1^ and 2890 cm^−1^ belong to –OH, –NH_2_, and aliphatic groups and absorption peaks at 1557 cm^−1^ depict the N–H bending vibrations. Broad peaks (at 3400 to 3500 cm^−1^) confirm the existence of the amine group in chitosan which is also overlapped with the broad peak of O–H. C–H bending vibration absorption at 1313 cm^−1^ and C–O in cellulose has an intense peak at 1253 cm^−1^ show the broad and red-shifted due to the bonding of AgNPs and oxygen atoms as shown in Fig. 2b. There is bond formation as N–X by halogenation of N–H groups. O–H bond (stretching vibration) in chitosan and cellulose molecules show absorption peaks at 3254 cm^−1^, C–H bond (stretching vibration) occurs at 2913 cm^−1^, and (C–H bending vibration) absorption peak occurs at 1388 cm^−1^. Stretching vibration of carbon and oxygen bond occurs at 1240 cm^−1^. The Amine group in chitosan has a particular absorption peak in the region from 3400 to 3500 cm^−1^. Absorption peaks at 1557 cm^−1^ decreased in intensity because after chlorination N–H bond converts into an N–Cl bond or the formation of *N-halamine* occurs. FTIR absorption spectrum of *N-halamine*-chitosan@AgNPs-nonwoven cotton fabric is shown below in Fig. 2c.

**Figure 3:**
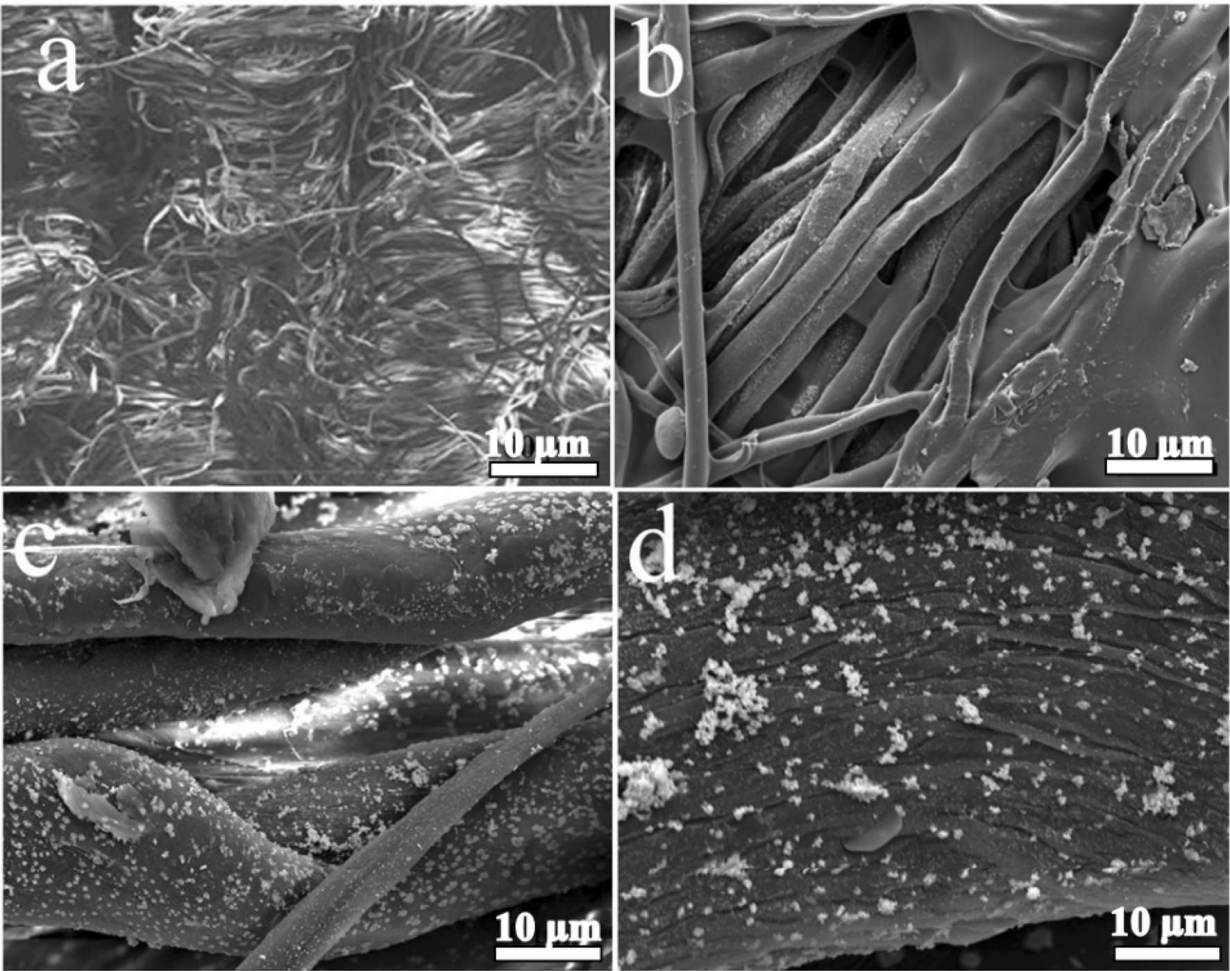
SEM images of non-woven cotton fabric (NWCF) (**a**), AgNPs-NWCF (**b**), Chitosan@AgNPs-NWCF (**c**), and  N-halamine-chitosan@AgNPs-NWCF (**d**).

AgNPs, chitosan, and *N-halamine*s modified fabric surface were further characterized by SEM. SEM image of nonwoven cotton fabric is shown below in Fig. 3a. AgNPs modified nonwoven cotton fabric show morphological changes. Some physical changes after the adsorption of silver nanoparticles on the surface of the fabric were also analyzed with SEM. It demonstrates that AgNPs coated NWFC has a smooth surface and uniform distribution of particles. SEM image of AgNPs-nonwoven cotton fabric is shown in Fig. 3b. SEM photograph of chitosan coating on AgNPs-nonwoven cotton fabric is clear. Crystallites are uniformly distributed. Accumulation of small particles can also be observed. SEM image of chitosan/AgNPs-nonwoven cotton fabric is shown in Fig. 3c. Loaded chlorine slightly alters the structure of the chitosan coating. Aggregation of tiny particles can also be observed. SEM image of *N-halamine*-chitosan@AgNPs-nonwoven cotton fabric is shown-Fig. 3d.

**Figure 4:**
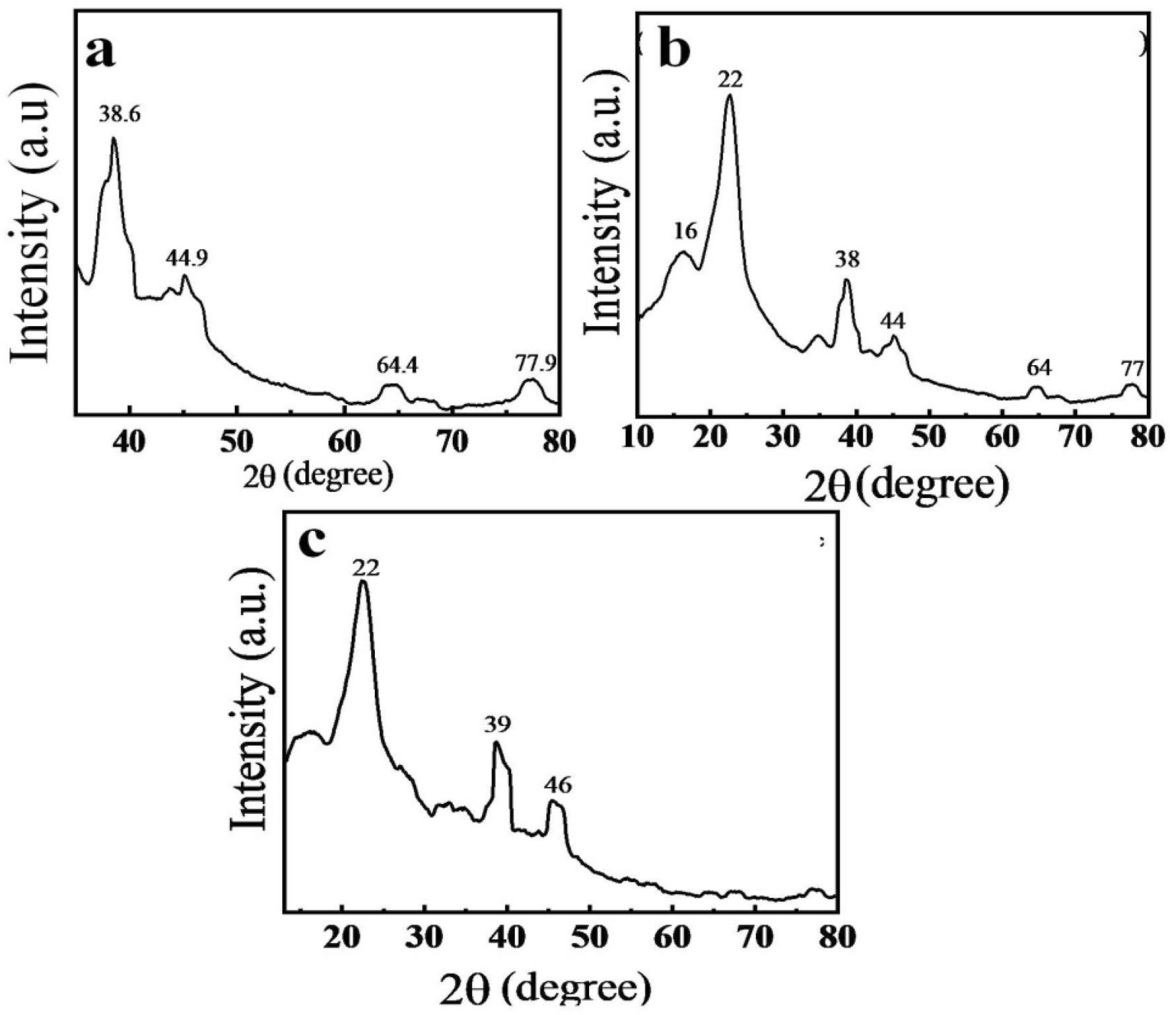
XRD patterns of AgNPs-nonwoven cotton fabric (**a**), Chitosan@AgNPs nonwoven cotton fabric (**b**), and *N-halamine*-chitosan@AgNPs-nonwoven cotton fabric (**c**).

XRD analysis of modified and unmodified nonwoven cotton fabric was carried out to observe the structural changes. An X-ray diffractometer was used for XRD analysis. Measurements were taken at 40 kV and 40 mA in 2θ range 10 to 80° with Cu-Kα radiation (*λ* = 0.15418 nm, 0.05 degrees *step size, 1 s per step*)^45,46^. XRD spectra of non-woven cotton fabric are shown in (Fig. 4). The peaks at 38.6°, 44.9°, 64.4°, and 77.9° obtained correspond to AgNPs-NWCF type represent the presence of silver in the fabric. (Fig. 4) These peaks belongs to silver nanoparticles are similar as reported earlier^47^.

Figure 4b for chitosan coating on AgNPs-NWCF shows characteristic peaks at 16° and 22° reveal chitosan with crystalline (a hydrated) structure^48^. A high-intensity peak at 22° represents the face-centered cubic material. X-ray diffraction pattern of chitosan@AgNPs- NWCF after the formation of N-halamine shows most prominent peaks at 22°, 39°, and 46° with small less intense peaks at more diffraction angles. Figure 4c The small peaks represent the small size nanoparticles^49^. All peaks are broad as compared to AgNPs-NWCF and chitosan@AgNPs-NWCF samples**.** It revealed that after chlorination of chitosan a new layer of chlorine atoms deposited and changes the diffraction angles. XRD pattern further indicates that the nonwoven samples, with and without modifiers, are free from major impurities, and no additional chemicals reactions take place after coatings.

### Preparation and characterization of antimicrobial coating on Nonwoven cotton fabric

**Figure 5:**
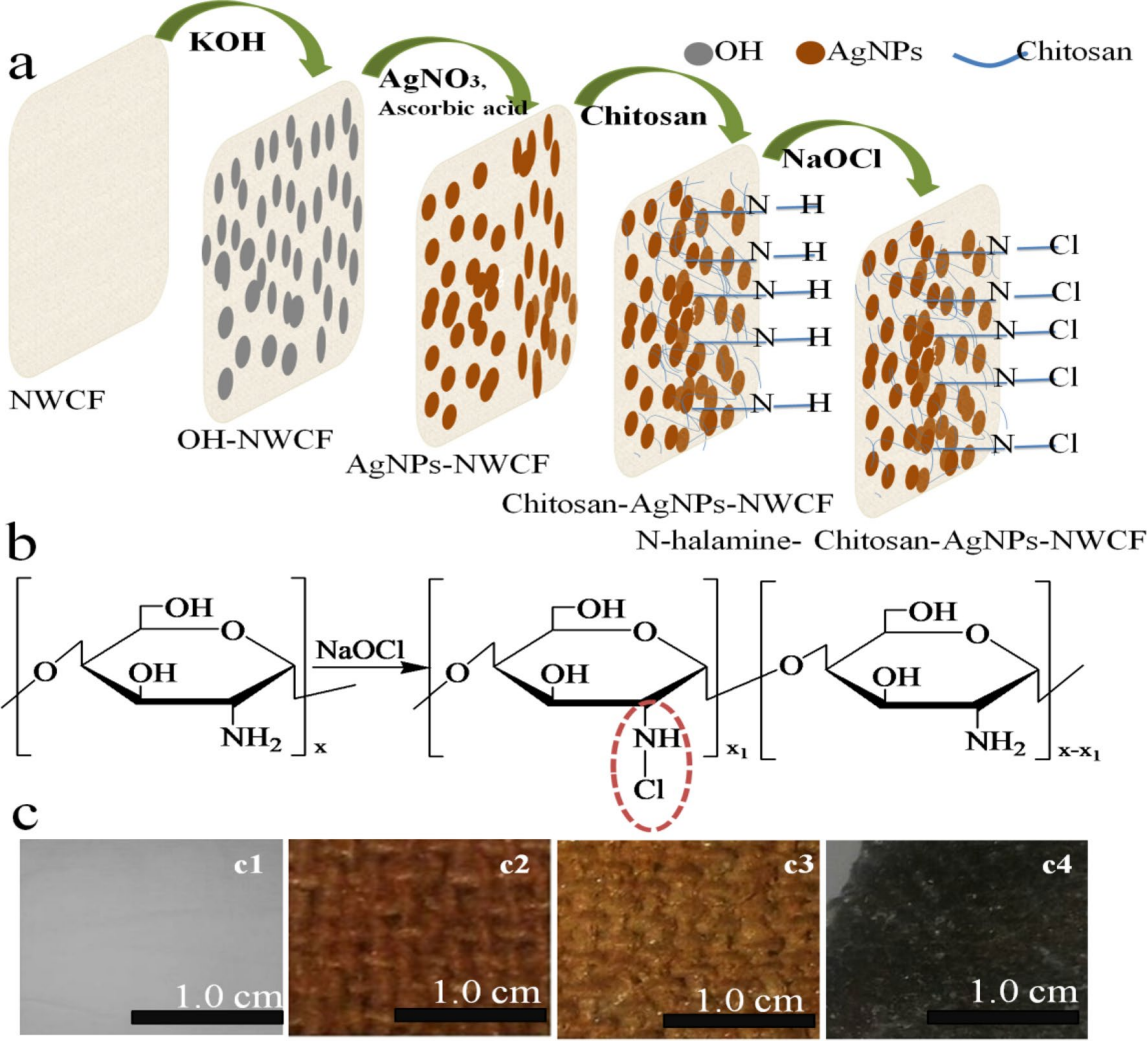
Modification and coating steps of nonwoven cotton fabric (**a**), Schematic illustration for halogenations of Chitosan (**b**) at fabric surface and visual color changes on fabric after coating and modification steps (**c**) photos, taken with camera , of non-woven cotton fabric (**c1**), the dark brown color obtained on modification with AgNPs (**c2**), the brown color obtained on chitosan coating (**c3**) and after *N-halamine* formation(**c4**). Loaded chlorine content analysis.

In industries, during fabric preparation, different types of chemicals or reagents are applied which protect it from insects, microbes, and other fungus attacks. The fabric surface is cleaned from residual chemicals before the surface modification process. In the present study, nonwoven fabric surfaces are modified using a dip-coating approach. The approach is simple, eco-friendly, cost-effective, and easy to execute. Surface roughness on the nonwoven cotton fabric is achieved with a 20 mL 6 M potassium hydroxide after cleaning with detergents and distilled water several times. A higher concentration of alkaline solution generated a higher number of active sites for further modification. Surface hydroxyl groups (OH) of the fabric cellulosic surface generate cellulose-OK^+^ on hydrolysis with potassium hydroxide. Cleaned and negatively charged surfaces of the fibers are further modified with in situ synthesis of silver nanoparticles. Alkali-treated cellulose-OK^+^ nonwoven fabric is dipped in silver nitrate solution. Reactive sites on the alkali-treated fabric are exchanged with Ag ions creating cellulose-OAg^+^. Silver ion modified surface is reduced further with ascorbic acid (C_6_H_8_O_6_) aqueous solution to generate silver nanoparticles (AgNPs) on the surface. After reduction of cellulose-OAg^+^ into AgNPs, fabric color changed from white to dark brown indicates the deposition of silver nanoparticles at fabric. Mechanism and schematic illustrations are shown in Fig. 5. *N-halamine* is the derivatization of chitosan polymeric materials with haloamine functional groups. This modification has attracted much attention in recent years due to its quick antibacterial response and vast potential in other biomedical and food packaging applications. Chitosan-AgNPs surfaces are positively charged that form covalent bonds with negatively charged chlorine from household bleach, requiring no additional surface modification. Therefore, the chitosan-AgNPs surface is modified with an *N-halamine* precursor simply by dipping.

Loaded chlorine content on AgNPs-NWCF, chitosan@AgNPs-NWCF and *N-halamine*s-chitosan@ AgNPs-NWCF is determined with simple iodine/thiosulphate titration. 1 mL sodium thiosulphate standard solution is used for 0.06 g (0.85 cm × 1.0 cm) of *N-halamine*s-chitosan@AgNPs-NWCF during titration. Whereas, AgNPs-NWCF (Control-I), chitosan@ AgNPs- NWCF (control-II) of same weight and dimensions consumed 0 mL sodium thiosulphate standard solution. Sodium thiosulphate volume is used to calculate the loaded chlorine content on chlorinated and unchlorinated fabrics, respectively^8,40,50^. Results indicate that *N-halamine*s-chitosan@ AgNPs-NWCF contain 0.295% chlorine whereas AgNPs- NWCF, chitosan@ AgNPs- NWCF contain 0% loaded chlorine concentration. The obtained results are shown in Table S1 (supplementary information).

### Antibacterial efficacy testing for *N-halamine*-chitosan@ AgNPs-NWCF

**Figure 6:**
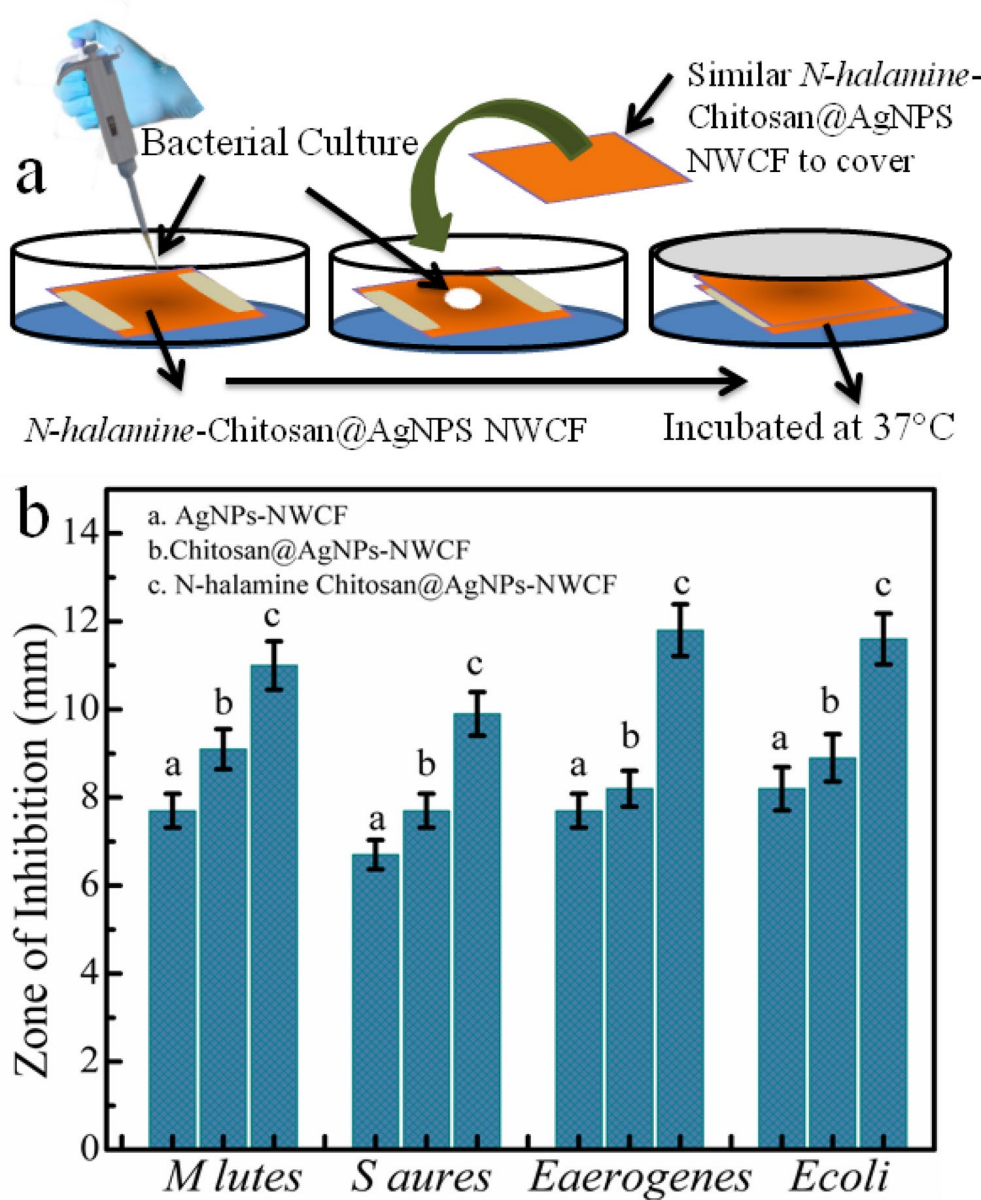
Schematic illustration (**a**) for measurement of antimicrobial efficacy of nonwoven cotton fabric, the response of growth inhibition zone (**b**) of AgNPs-NWCF (Control-I), chitosan@AgNPs-NWCF (Control-II), and *N-halamine*s-chitosan@AgNPs-NWCF (Sample) for four types of bacteria. An error bar has been added for n = 3. The trend line at bars shows bacterial response behavior for AgNPs-NWCF (control I), chitosan@AgNPs-NWCF (II), and *N-halamine*s-chitosan@AgNPs-NWCF (Sample).

Biocidal properties of AgNPs- NWCF, chitosan@ AgNPs- NWCF, and *N-halamine*s-chitosan@ AgNPs-NWCF samples were tested against four types of bacteria: *Micrococcus luteus* (Gram-positive), *Staphylococcus aureus* (Gram-positive), *Enterobacter aerogenes* (Gram-negative), and *E.coli* (Gram-negative) following established procedures^40^ as detailed in material and methods section and schematically in Fig. 6a.

The fabric samples were exposed to bacterial strains at 1.7 × 10^5^ CFU / fabric patch. Figure 6b response graph and Table S2 (supplementary information) show the antibacterial results. The trend lines reveals that *N-halamine*s-chitosan@ AgNPs-NWCF offers highest inhibition zone in bacterial strains among the executed materials. The samples of AgNPs-NWCF and chitosan@AgNPs-NWCF chitosan offered low bactericidal activity against *Micrococcus luteus, Staphylococcus aureus, Enterobacter aerogenes,* and *E.coli strains.* The log reductions of 6.7, 6.6, 5.6, 5.4, and 7.8, 7.7, 7.5, 7.3, respectively were observed for the aforementioned bacterial strains. The modified fabric samples provided about 11.2 to 11.9 log reduction within 15 min of exposure time compared to control.

**Figure 7:**
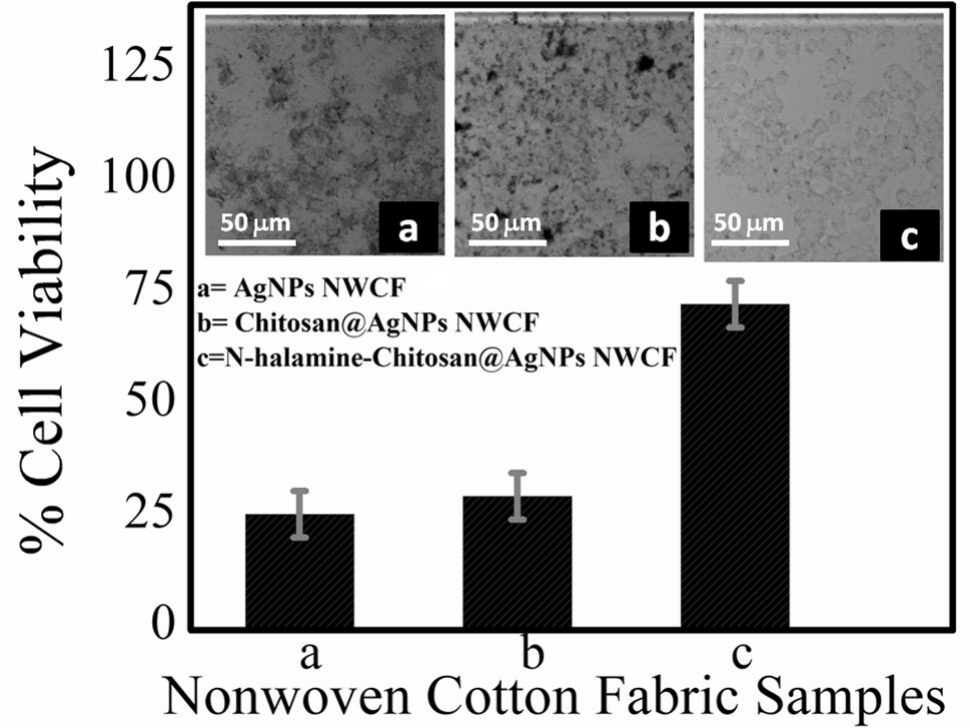
Cell viability analysis (MTT assay) on *Hela Cell* lines of AgNPs-NWCF, Chitosan@AgNPs-NWCF, *N-halamine-*Chitosan@AgNPs-NWCF. Inside images show confocal microscopic cell images.

The inhibition efficacies of the *N-halamine*-chitosan@ AgNPs-NWCF improved significantly compared with other samples. The reduction in the number of bacteria in media is attributed to the attachment of bacteria to the fabric samples and the inactivation by the *N-Halamine* and silver nanoparticles modified fabric. It is assumed that the inactivation of bacterial growth has been achieved through the transfer of positive halogen (Cl^+^) from *N-halamine* coating to the growth medium. In addition, *N-halamine* containing stable N–X bond as shown in XRD and FTIR pattern also tends to add killing effect through contact with fabric surface^9,51–53^. More interestingly, it is proposed that the antibacterial activity cannot be explained alone due to halogen release or contact but also combination effect of silver nanoparticles and *N-halamine* simultaneously. The results are similar to previous reports with the inhibition zone of 11.2 mm, 9.8 mm, 11.9 mm and 11.8 mm N*-halamine*-chitosan@ AgNPs-NWCF samples for *Micrococcus luteus, Staphylococcus aureus, Enterobacter aerogenes,* and *E.coli* strains, respectively^54^. The *N-halamine*-chitosan@ AgNPs-NWCF samples inhibit bacterial growth, with lower bacterial activity for Gram‐negative bacteria than Gram‐positive bacteria. This fact is attributed to the different shapes, sizes, surface structures, and more resistance to the inactivation of bacteria over other strains^40^. The results are equal to or consistent with the previous reports^8,50,55,56^. Cytotoxicity studies of *N-halamine*-chitosan@AgNPs-NWCF The effect of modifiers N-halamine, chitosan, and silver nanoparticles on cell survivability on the *Hela cells* was evaluated Fig. 7. The cell viability analyses tell about the number of living cells remaining after treatment as a percentage cell viability against positive control. In the present study, cell viability % was calculated for AgNPs-NWCF, chitosan@AgNPs-NWCF, *N-halamine*-chitosan@AgNPs-NWFC samples against positive control. Positive controls were considered 100% cell viable as they contained no modified NWCF material. Cell viability of NWCF materials was evaluated against positive control. *N-halamine*-treated Chitosan@AgNPs-NWFC samples showed cell viability by up to 85%. These results suggested that N-halamine modified nonwoven cotton fabric does not significantly inhibit cell viability. The deviation in percentage from 100% value may be attributed to the Cl^+^ ions released from the fabric surface and might have interfered with the cell viability measurements. Compared to *N-halamine-*Chitosan@AgNPs-NWFC, AgNPs-NWCF, Chitosan@AgNPs-NWCF showed 25% and 30% cell viability, respectively, which shows their significant toxicity towards *HeLa cell* Lines. These results suggest that *N-halamine* functionalization leads to decrease, rather than increase, in cell toxicity of NWCF. ; the results are in agreement with other reported results for similar studies^42,43,50,57^. However, it is also assumed that the small toxicity observed in the present experiment may be due to the release or dissociation of Cl^+^ into cell medium with time from *N-halamine* modified chitosan@AgNPs-NWFC. The release can be controlled by optimizing the chlorine contents at nonwoven cotton fabric and with repeated washing with water without affecting fabricated fabric properties.

### Photostability and shelf life stability

Nonwoven cotton fabric samples are investigated for Photostability and shelf life at room temperature. It has been reported in the literature that N–Cl bonds in N-*halamine* are sensitive to light irradiations. The dissociation of bonds increased with exposure to UVA light, indicating the dissociation of the N–Cl bond and releasing Cl_2_^58^. The shelf life (storage) stability of bound chlorine in *N-halamine*s-chitosan@AgNPs-nonwoven fabric under UVA lamp and dark environment for 12 weeks are shown in Figure S1 (Supplementary Information). *N-halamine*s-chitosan@AgNPs-NWCF stored in dark environmental conditions retained most of their initial chlorine loadings for 6 weeks. *N-halamine*s-chitosan@AgNPs-NWCF lost only 20% of chlorine after 12 weeks of storage in darkness. It is observed that *N-halamine*s-chitosan@AgNPs-NWCF has excellent storage stability and retained 80% ± 10 of their chlorine content after 12 weeks in a dark environment. However, *N-halamine*s-chitosan@AgNPs-NWCF is somewhat less stable under light. It is noted that the N–Cl bond dissociation increased with exposure to UVA light. When stored under light, *N-halamine*s-chitosan@AgNPs-NWCF samples lost only 30% of the oxidative chlorine over 12 weeks of storage. Photostability of *N-halamine* modified data is comparable with previous reports^23^.

## Conclusion

A cost-effective and eco-friendly nonwoven cotton fabric with antibacterial and nontoxic properties is fabricated in this study. N-halamine and silver nanoparticles have been coated onto nonwoven cotton fabric samples using the dip-coating method. The FTIR, SEM, and XRD analyses were used to characterize the modified surface. The analysis showed successful coating and functionalization onto nonwoven cotton fabric, and not damage under the acidic conditions of treatment. The antibacterial test showed a significant decrease in the bacterial colony on exposure to N*-halamine*s- chitosan @AgNPs-nonwoven cotton fabric as compared to AgNPs-nonwoven cotton fabric, chitosan@AgNPs-nonwoven cotton fabric. The addition of N*-halamine*s onto samples increases the antibacterial activity of gram-positive bacteria than gram-negative bacteria. Moreover, the N*-halamines* modified chitosan @AgNPs-nonwoven cotton fabric showed less cell toxicity against HeLa cell lines as compared to AgNPs-nonwoven cotton fabric and chitosan@AgNPs-nonwoven cotton fabric. It can be concluded that the present modification method is stable, simple, low cost, and environmentally friendly yet to be established in laboratories for various biomedical applications. Furthermore, *N-halamine*-chitosan @AgNPs-nonwoven cotton fabric may have a wide range of applications in biomedicine and health care, including wound dressings, surgical masks, coatings for medical devices, and hospital linens.

## Supplementary Information

Supplementary Information.
