# Supplementary material for: Cost-effective fabrication, antibacterial application and cell viability studies of modified nonwoven cotton fabric
Source: Sci Rep. 2022 Feb 15;12:2493. doi: 10.1038/s41598-022-06391-5 (PMC8847346; doi:10.1038/s41598-022-06391-5)
Supplement: Supplementary file 1 — Supplementary Information. [file 41598_2022_6391_MOESM1_ESM.docx]

**Cost-Effective Fabrication, Antibacterial Application and Cell Viability Studies of Modified Nonwoven Cotton Fabric**

Rahat Nawaz,^a^ Sayed Tayyab Raza Naqvi,^a^ Batool Fatima,^b^ Nazia Zulfiqar, ^a^ Muhammad Umar Farooq, ^a^ Muhammad Najam ul Haq, ^a^  Dilshad Hussain,^c^ Asghar Javeed, ^d^ Azhar Rasul,^e^ Laila Jafri,^f^ Waheed Qamar Khan,^g^ Saadat Majeed ⃰ ^a^

^a^Division of Analytical Chemistry, Institute of Chemical Sciences, Bahauddin Zakariya University, Multan 60800, Pakistan

^b^Department of Biochemistry, Bahauddin Zakariya University, Multan 60800, Pakistan

^c^ International Centre for Chemical and Biological Sciences, HEJ Research Institute of Chemistry, University of Karachi, Karachi 75270, Pakistan

^d^Department of Pathology, Nishtar Medical University, Multan 60800, Pakistan

**^e^** Department of Zoology, Govt. College University Faisalabad, Faisalabad

^f^ Department of Life sciences, Abasyn University, Islamabad Campus, Islamabad Pakistan

**^g^ Institute of Advanced Materials,** Bahauddin Zakariya University, Multan 60800, Pakistan

^*^E-mail:[saadat.majeed@bzu.edu.pk](mailto:saadat.majeed@bzu.edu.pk)

[**Figure S1**](https://www.ncbi.nlm.nih.gov/pmc/articles/PMC6151735/table/molecules-22-01582-t001/?report=objectonly)**. Photostability and shelf-life stability of *N-halamine*s-chitosan@AgNPs-nonwoven fabric under dark and UVA light conditions for upto 12 weeks. Error bars added for N=3 replicates.**

**

**

**Table S1. Percentage loading of chlorine on Non-Woven Cotton Fabric samples**

| **Samples** | **Non-Woven Cotton Fabric** | **Weight** | **Chitosan**  **(2% sol.)** | **Chlorination Time**  **(mint.)** | **Volume**  **(mL)** | **Loaded chlorine**  **(%)** |
| --- | --- | --- | --- | --- | --- | --- |
| AgNPs-NWCF  (0.85 cm ×1.0cm) | Unchlorinated  (control-I) | 0.06g | - | - | 0.01 | 0 |
| Chitosan@ AgNPs-NWCF(0.85 cm ×1.0cm) | Unchlorinated  (control-II) | 0.06g | Double  coating | - | 0.01 | 0 |
| *N-halamine*/ chitosan@ AgNPs-NWCF  (0.85 cm ×1.0cm) | Chlorinated | 0.06g | Double  coating | 30 | 0.01 | 0.295 |

**Antibacterial Testing**

Bacterial reduction (%) = (B- A)/ B × 100 %

Where, A is the number of surviving bacterial colonies of the test sample and B is that of the control.

**Table S2. Growth inhibition zone of AgNPs-nonwoven fabric, chitosan@AgNPs-nonwoven fabric, and *N-halamine*s-chitosan@AgNPs-nonwoven fabric for *Micrococcus lutes, Staphylococcus aureus, Enterobacter aerogenes* and *E.coli bacteria. Experiments were performed in triplicate.***

| **Sr.** | **Sample** | **Zone of inhibition with 6mm solid disk sample** | | | |
| --- | --- | --- | --- | --- | --- |
|  |  | ***Micrococcus lutes***  ***mm***±SD | ***Staphylococcus***  ***Aureus***  ***mm***±SD | ***Enterobacter***  ***Aerogenes***  ***mm***±SD | ***Escherichia***  ***Coli***  ***mm***±SD |
| 1 | AgNPs-NWCF | 6.7±1.3 | 6.6±1.21 | 5.6±1.00 | 5.4±1.00 |
| 2 | Chitosan/@AgNPs-NWCF | 7.8±0.35 | 7.7±0.01 | 7.5±0.23 | 7.3±0.22 |
| 3 | *N-halamine*s - chitosan@AgNPs-NWCF | 11.8±1.1 | 11.9±0.02 | 11.2±0.02 | 11.60±0.15 |
